# Introducing new and effective catalysts for the synthesis of pyridazino[1,2-*a*]indazole, indazolo[2,1-*b*]phthalazine and pyrazolo[1,2-*b*]phthalazine derivatives

**DOI:** 10.1016/j.mex.2020.100823

**Published:** 2020-02-20

**Authors:** Najmeh Amirmahani, Nosrat O. Mahmoodi, Mohammad Malakootian, Abbas Pardakhty

**Affiliations:** a Department of Chemistry, University Campus 2, University of Guilan, Rasht, Iran; b Environmental Health Engineering Research Center, Kerman University of Medical Sciences, Kerman, Iran; c Department of Chemistry, Faculty of Science, University of Guilan, Rasht, Iran; d Pharmaceutics Research Center, Neuropharmacology Institute, Kerman University of Medical Sciences, Kerman, Iran

**Keywords:** Four-component condensation reaction, Synthesis, Catalyst, Pyridazino[1,2-*a*]indazole, Indazolo[2,1-*b*]phthalazine, Pyrazolo[1,2-*b*]phthalazine, Triethanolammonium acetate ([TEAH][OAc])

## Abstract

In this study, a new and effective catalyst for the synthesis of pyridazino[1,2-*a*]indazole, indazolo[2,1-*b*]phthalazine and pyrazolo[1,2-*b*]phthalazine derivatives was introduced. Triethanolammonium acetate ([TEAH][OAc]) accelerates the reaction in a one-pot and four-component condensation of aldehydes, hydrazine hydrate, succinic/phthalic anhydride, and 1,3-dicarbonyl compounds. The yield of the products is high, and the reaction conditions are mild and solvent-free. Furthermore, the model reaction was conducted in the presence of triethanolammonium sulphate ([TEAH][HSO_4_] and triethanolammonium formate ([TEAH][HCOO]) under various conditions. In addition, the catalyst is recyclable, therefore, it can be reused several times. The structure of the obtained products was confirmed by comparing the M.P., IR, and ^1^H NMR.

Advantages of this technique are as following:

• Synthesis of novel, green, and one-pot and four-component condensation (4CC) under solvent-free conditions at room temperature.
• The catalytic reaction is performed under mild and environmentally friendly conditions in short reaction times and excellent yields.
• The catalyst is easily recycled and exhibits good chemical and structural stability.

## Graphical abstract

**Figure:**
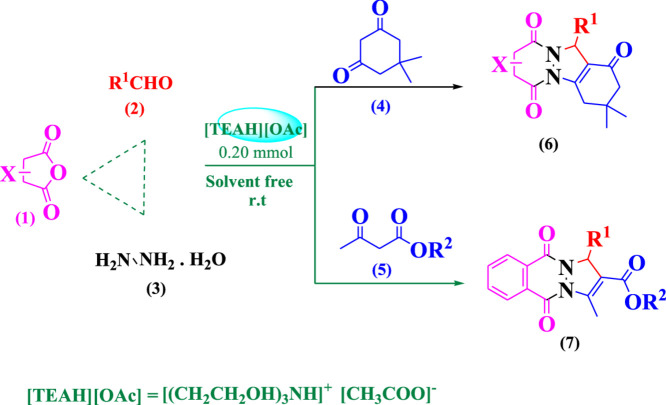

**Table:** Specification table

Subject Area:	*Chemistry*
More specific subject area:	*Organic Chemistry*
Method name:	*Ionic liquid as a catalyst for the synthesis of heterocyclic compounds*
Name and reference of original method:	Direct submission
Resource availability:	Direct submission

## Methods

Despite recent advances in molecular biology and synthetic combinatorial methodology, the rate of introduction of new drugs has significantly declined over the past two decades. It is believed that making diversity in a potential therapeutic complex increases the rate of success. Most of the drugs that are still in use, are synthetic small organic molecules, often containing a heterocyclic ring. However, a range of easily accessible heterocyclic structures with functional groups suitable for the synthesis of diverse structures in the laboratory is limited. Therefore, the development of new, rapid, and accurate synthetic pathways for these heterocyclic compounds in the laboratory has been very important for the pharmaceutical and synthetic chemists. Undoubtedly, the most efficient tool involves multicomponent reactions (MCRs), which is a powerful tool for the rapid production of diverse compounds [1]. As a result, the design and development of MCRs have received much attention. Multicomponent reactions are a specific type of synthetic useful organic reactions, in which three or more raw materials react to produce the final product in a one-pot method. MCRs are a powerful tool for discovering new drugs, enabling the rapid and automatic production of high-efficiency organic compounds. In addition, the discovery of new MCRs can be considered as an interesting subject for academic research [2].

The development of MCRs in heterocyclic synthesis has attracted the attention of many chemists to synthesize pharmacological compounds. One of the widespread applications of these reactions is the synthesis of indazoles and pyrazoles derivatives.

Indazoles and pyrazoles derivatives exhibit a wide range of biological and pharmacological activities, such as the inhibition of protein kinase C-β [3], 5-HT2 and 5-HT3 receptor antagonisms [4], ability to bind to estrogen receptor [5], and HIV virus inhibition [6]. In organic chemistry, there are various uses of solvents. These solvents affect the living organisms because of their toxic nature, which is highly permeable to the environment. To avoid the use of such toxic solvents, the reactions can be carried out using catalysts such as ionic liquids or biological catalysts, which do not harm the environment [7,8]. With increasing the community awareness about recyclable compounds, bio-based and environmentally friendly products have taken a higher priority. Conventional catalysts, such as H_2_SO_4_ and HCl, which are acidic catalysts, or alkaline catalysts, such as NaOH, can be replaced by bio-friendly and environmentally friendly catalysts, such as ionic liquids, which act as both acidic and alkaline catalysts. The main goal of green chemistry is to achieve higher efficiency with lower waste and avoid the use of toxic solvents [9,10]. In recent years, ionic liquids have become strong organic solvents because of their special properties, such as ease of product recovery and catalyst recycling [[12], [13], [14], [15], [16], [11]].

**Scheme 1:**
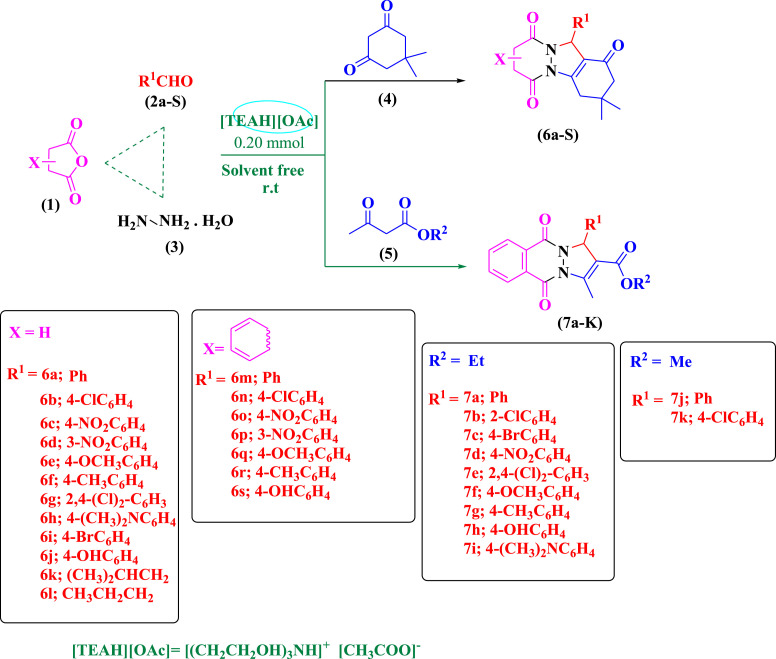
[TEAH][OAc] catalyzed the synthesis of 6a-7k products.

Following on from our previous work [17], the present study focused on an easy and green way to synthesize 2*H*-pyridazino[1,2-*a*]indazole-1,6,9(11*H*)-triones, 2*H*-indazolo[2,1-*b*]phthalazine-1,6,11(13*H*)-triones, and 1*H*-pyrazolo[1,2-*b*]phthalazine-2-carboxylate derivatives under solvent-free conditions. For this purpose, ([TEAH][OAc]) was used as an ionic liquid catalyst to promote the reaction (Scheme 1). Then, in the other efforts ([TEAH][HSO4] and ([TEAH][HCOO]) were used as ionic liquid catalysts to promote the model reaction (Table 1).

**Table 1:** Determination of optimal reaction conditions^a^.


Entry	Catalyst (mmol)	Conditions	Time	Yield^b^ (%)
1	–	Solvent-Free (100 °C)	24 (h)	0
2	[TEAH][OAc] (0.05 mmol)	Solvent-Free (r.t.)	24 (h)	10
3	[TEAH][OAc] (0.05 mmol)	Solvent-Free (50 °C)	24 (h)	25
4	[TEAH][OAc] (0.05 mmol)	Solvent-Free (80 °C)	12 (h)	20
5	[TEAH][OAc] (0.10 mmol)	Solvent-Free (50 °C)	5 (h)	30
6	[TEAH][OAc] (0.10 mmol)	Solvent-Free (80 °C)	4 (h)	40
7	[TEAH][OAc] (0.10 mmol)	Solvent-Free (100 °C)	3 (h)	40
8	[TEAH][OAc] (0.20 mmol)	Solvent-Free (30 °C)	1 (h)	40
9	[TEAH][OAc] (0.20 mmol)	Solvent-Free (50 °C)	40 (min)	80
10	[TEAH][OAc] (0.20 mmol)	Solvent-Free (80 °C)	20 (min)	91
11	[TEAH][OAc] (0.20 mmol)	Solvent-Free (100 °C)	20 (min)	91
12	[TEAH][OAc] (0.25 mmol)	Solvent-Free (80 °C)	20 (min)	91
14	[TEAH][OAc] (0.15 mmol)	EtOH (80 °C)	100 (min)	20
15	[TEAH][OAc] (0.15 mmol)	EtOH/H_2_O (80 °C)	120 (min)	25
16	[TEAH][OAc] (0.15 mmol)	H_2_O (80 °C)	175 (min)	20
17	[TEAH][OAc] (0.15 mmol)	THF (80 °C)	220 (min)	Trace
18	[TEAH][HSO_4_] (0.10 mmol)	Solvent-Free (70 °C)	70 (min)	52
19	[TEAH][HSO_4_] (0.20 mmol)	Solvent-Free (80 °C)	70 (min)	69
20	[TEAH][HSO_4_] (0.25 mmol)	Solvent-Free (80 °C)	75 (min)	70
21	[TEAH][HSO_4_] (0.20 mmol)	Solvent-Free (90 °C)	70 (min)	75
22	[TEAH][HSO_4_] (0.20 mmol)	Solvent-Free (100 °C)	70 (min)	75
23	[TEAH][HCOO] (0.10 mmol)	Solvent-Free (70 °C)	65 (min)	60
24	[TEAH][HCOO] (0.20 mmol)	Solvent-Free (80 °C)	55 (min)	78
25	[TEAH][HCOO] (0.25 mmol)	Solvent-Free (80 °C)	55 (min)	79
26	[TEAH][HCOO] (0.20 mmol)	Solvent-Free (90 °C)	55 (min)	78
27	[TEAH][HCOO] (0.20 mmol)	Solvent-Free (100 °C)	55 (min)	78

a Reaction conditions: 1 (1 mmol), 2 (benzaldehyde, 1 mmol), 3(1.1 mmol) and 4(1 mmol).

b Yields refer to pure isolated yields.

### Experimental design, materials, and methods

Initially, the production of 6a was considered as the model reaction, and formerly, changes of solvent, temperature, and catalyst content were investigated. The indazoles and pyrazoles derivatives were prepared by the one-pot and four-component condensation (4CC). It was focused on a one-pot, 4CC of aldehydes, hydrazine hydrate, succinic/phthalic anhydride, and 1,3-dicarbonyl compounds in the absence or presence of different catalysts under various conditions, and the results are listed in Table 1. In the absence of any catalysts, the model reaction did not proceed successfully and no corresponding product was prepared (Table 1, entry 1). The model reaction was conducted in the presence of [TEAH][OAc] under various conditions (Table 1, entries 1–17) and the best results was obtained in the presence of 20 mol% [TEAH][OAc] under solvent-free condition at 80 °C (Table 1, entry 10). Furthermore, in the presence of ionic liquid [TEAH][HSO4], the desired product was obtained in 52–75% yield (Table 1, entries 18–22) and in the presence of ionic liquid [TEAH][HCOO], the desired product was obtained in 60–79% yield (Table 1, entries 23–27).

In the presence of ionic liquid catalyst [TEAH][OAc] the desired products with high efficiency and purity was obtained under solvent-free conditions. The structures of the products were confirmed by M.P., IR, ^1^H NMR data and comparison with those of authentic samples that obtained earlier.

All the samples synthesized under optimal conditions are listed in Table 2.

**Table 2:** Products synthesized by [TEAH][OAc] catalyst under optimal conditions^a^.

Entry	*R*^1^	*R*^2^	1,3-Dicarbonyl	Product	Time (min)	Yield^b^^,^^c^ (%)
1	pH	–	Dimedone	6a	20	91 [18]
2	4-ClC_6_H_4_	–	Dimedone	6b	18	92 [18]
3	4-O_2_NC_6_H_4_	–	Dimedone	6c	15	88 [18]
4	3-O_2_NC_6_H_4_	–	Dimedone	6d	18	82 [18]
5	4-CH_3_OC_6_H_4_	–	Dimedone	6e	30	90 [18]
6	4-CH_3_C_6_H_4_	–	Dimedone	6f	25	70 [18]
7	2,4-(Cl)_2_—C_6_H_3_	–	Dimedone	6g	20	93 [18]
8	4-(CH_3_)_2_NC_6_H_4_	–	Dimedone	6h	20	88 [18]
9	4-BrC_6_H_4_	–	Dimedone	6i	22	79 [18]
10	4-HOC_6_H_4_	–	Dimedone	6j	30	80 [18]
11	(CH_3_)_2_CHCH_2_	–	Dimedone	6k	40	57 This Work
12	CH_3_CH_2_CH_2_	–	Dimedone	6l	35	50 This Work
13	pH	–	Dimedone	6m	13	91 [19]
14	4-ClC_6_H_4_	–	Dimedone	6n	12	90 [19]
15	4-O_2_NC_6_H_4_	–	Dimedone	6o	10	89 [19]
16	3-O_2_NC_6_H_4_	–	Dimedone	6p	13	95 [19]
17	4-CH_3_OC_6_H_4_	–	Dimedone	6q	20	92 [19]
18	4- CH_3_C_6_H_4_	–	Dimedone	6r	35	92 [19]
19	4-HOC_6_H_4_	–	Dimedone	6s	20	91 [19]
20	PhCHO	Et	Ethyl 3-oxobutanoate	7a	37	82 [19]
21	2-ClC_6_H_4_	Et	Ethyl 3-oxobutanoate	7b	32	89 [20]
22	4-BrC_6_H_4_	Et	Ethyl 3-oxobutanoate	7c	35	92 [20]
23	4-NO_2_C_6_H_4_	Et	Ethyl 3-oxobutanoate	7d	22	95 [20]
24	2,4-Cl, Cl C_6_H_4_	Et	Ethyl 3-oxobutanoate	7e	30	87 [20]
25	4-OCH_3_C_6_H_4_	Et	Ethyl 3-oxobutanoate	7f	35	78 [20]
26	4-CH_3_C_6_H_4_	Et	Ethyl 3-oxobutanoate	7g	35	75 [20]
27	4-OHC_6_H_4_	Et	Ethyl 3-oxobutanoate	7h	28	80 [20]
28	4-N(CH_3_)_2_C6H4	Et	Ethyl 3-oxobutanoate	7i	36	78 [20]
29	pH	Et	methyl 3-oxobutanoate	7j	35	85 [20]
30	4-ClC_6_H_4_	Et	methyl 3-oxobutanoate	7k	30	87 [20]

a Reaction conditions: 1 (1 mmol), 2 (1 mmol), 3 (1.1 mmol), 4 or 5 (1 mmol) and [(CH_2_CH_2_OH)_3_NH][CH_3_COO] (0.20 m mol) (Scheme 1) solvent-free, 80 °C.

b Yields refer to pure isolated yields.

c Reference numbers for known compounds.

### Stability and recycling of the catalyst

To promote green synthesis in organic chemistry, the catalyst stability and its reuse were investigated. For this purpose, after the completion of the reaction, the catalyst was separated. The aqueous layer containing the catalyst was separated and extracted under reduced pressure of evaporated water. The obtained ionic liquid was reused in the model reaction. The results showed that the [TEAH][OAc] catalyst was reusable up to the fourth time without a significant decrease in the activity. On the other hand, during four stages of the reaction efficiency recovery, the model was 91% in the first stage and 86% in the fourth stage, indicating that the catalytic activity did not decrease significantly.

**Scheme 2:**
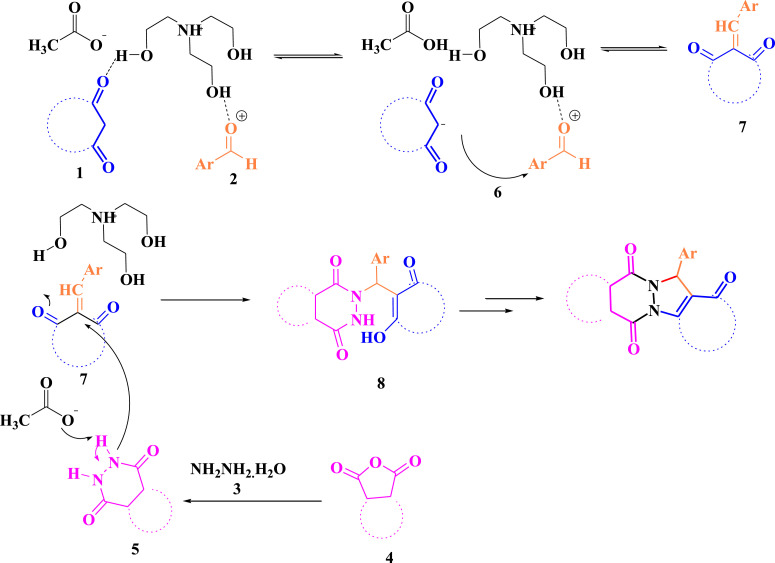
The proposed mechanism for the synthesis of 6a using [TEAH][OA*c*].

The proposed mechanism for the synthesis of 6a-7k derivatives by the [TEAH][OAc] catalyst is shown in Scheme 2.

Firstly, it was assumed that the reaction is conducted via a Knoevenagel condensation between 1,3-dicarbonyl compound **1** and aromatic aldehyde **2** to form the intermediate **7** via intermediate 6 in the presence of [TEAH][OA*c*], that undergoes immediate Michael addition via C=C bond of **7**. During the reaction process, the hydrogen ion is transferred from the [TEAH][OAc] to intermediate **8**. The concerted cyclocondensation of the amine and carbonyl of the Michael lead to the corresponding product **8** (Scheme 2).

## Conclusion

**Fig. 1:**
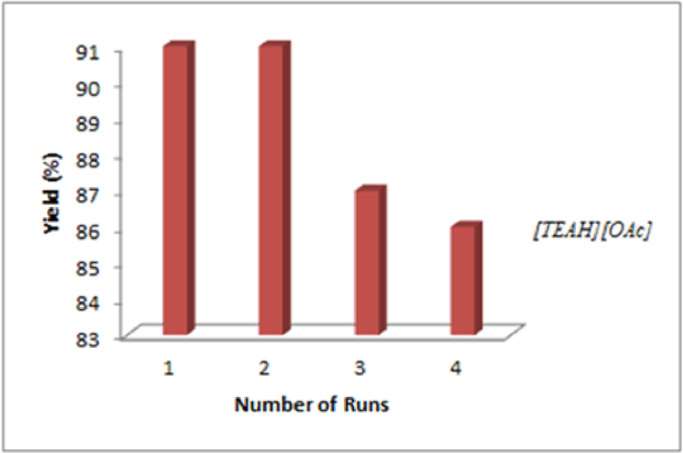
Recyclability of the catalyst in the model reaction under optimal reaction conditions.

In this research, new, green, and effective catalysts for the synthesis of pyridazino[1,2-*a*]indazole, indazolo[2,1-*b*]phthalazine, and pyrazolo[1,2-*b*]phthalazine derivatives by one-pot 4CC were used. These catalysts have advantages, such as reduced reaction time, high product efficiency, ease of use, and recycling (Fig. 1).

## Declaration of Competing Interest

The Authors confirm that there are no conflicts of interest.
